# Nonsynonymous substitution rate (Ka) is a relatively consistent parameter for defining fast-evolving and slow-evolving protein-coding genes

**DOI:** 10.1186/1745-6150-6-13

**Published:** 2011-02-22

**Authors:** Dapeng Wang, Fei Liu, Lei Wang, Shi Huang, Jun Yu

**Affiliations:** 1 CAS Key Laboratory of Genome Sciences and Information, Beijing Institute of Genomics, Chinese Academy of Sciences, Beijing 100029, PR China; 2 Graduate University of Chinese Academy of Sciences, Beijing 100049, PR China; 3 State Key Laboratory of Medical Genetics, Xiangya Medical School, Central South University, Changsha, Hunan 410078, PR China

## Abstract

**Background:**

Mammalian genome sequence data are being acquired in large quantities and at enormous speeds. We now have a tremendous opportunity to better understand which genes are the most variable or conserved, and what their particular functions and evolutionary dynamics are, through comparative genomics.

**Results:**

We chose human and eleven other high-coverage mammalian genome data–as well as an avian genome as an outgroup–to analyze orthologous protein-coding genes using nonsynonymous (Ka) and synonymous (Ks) substitution rates. After evaluating eight commonly-used methods of Ka and Ks calculation, we observed that these methods yielded a nearly uniform result when estimating Ka, but not Ks (or Ka/Ks). When sorting genes based on Ka, we noticed that fast-evolving and slow-evolving genes often belonged to different functional classes, with respect to species-specificity and lineage-specificity. In particular, we identified two functional classes of genes in the acquired immune system. Fast-evolving genes coded for signal-transducing proteins, such as receptors, ligands, cytokines, and CDs (cluster of differentiation, mostly surface proteins), whereas the slow-evolving genes were for function-modulating proteins, such as kinases and adaptor proteins. In addition, among slow-evolving genes that had functions related to the central nervous system, neurodegenerative disease-related pathways were enriched significantly in most mammalian species. We also confirmed that gene expression was negatively correlated with evolution rate, i.e. slow-evolving genes were expressed at higher levels than fast-evolving genes. Our results indicated that the functional specializations of the three major mammalian clades were: sensory perception and oncogenesis in primates, reproduction and hormone regulation in large mammals, and immunity and angiotensin in rodents.

**Conclusion:**

Our study suggests that Ka calculation, which is less biased compared to Ks and Ka/Ks, can be used as a parameter to sort genes by evolution rate and can also provide a way to categorize common protein functions and define their interaction networks, either pair-wise or in defined lineages or subgroups. Evaluating gene evolution based on Ka and Ks calculations can be done with large datasets, such as mammalian genomes.

**Reviewers:**

This article has been reviewed by Drs. Anamaria Necsulea (nominated by Nicolas Galtier), Subhajyoti De (nominated by Sarah Teichmann) and Claus O. Wilke.

## Background

Although protein-coding sequences account for ~1% of the entire mammalian genome, it is the most function-related, dynamic, and informative part of the genome [1]. For molecular evolution studies, protein-coding sequences are central to understanding the mutational dynamics of genes and the functional dynamics of gene networks within a population or among diverse species and lineages.

Following the publication of the complete human genome sequence [2], over a dozen mammalian genomes have been sequenced, allowing mammalian comparative genomics to finally come to age. Genome-wide sequence analysis has been focused on two essential forms of genetic variation. One concerns gene gain-and-loss that is related to the amplification and deletion of certain genes and their chromosomal regions. This is an important evolutionary mechanism to shape mammalian genomes through natural selection, but it also leads to gene family expansion and deletion, which has been proposed to be one molecular origin of chimp-human evolution [3]. Another form of genetic variation is sequence variation at specific nucleotide sites in protein-coding genes. Such variations become functionally relevant when they alter protein sequences.

The task of defining positively-selected genes has drawn the most attention, because these genes are often considered to be the major driving forces behind how organisms adapt to their external environments [4,5]. A number of interesting characteristics of positively selected genes have been found: (1) they are more likely to have several classes of functions, including nuclear transport, sensory perception, immune defenses, tumor suppression, apoptosis, and reproduction, and may be involved in Mendelian genetic disorders [6-8]. (2) These genes tend to be expressed at low levels and in a tissue-specific manner [7]. (3) Some highly-expressed genes in the testis were reported to have been subjected to positive selection [6]. (4) Positively selected genes are often species-specific or lineage-specific [7]. As the number of sequenced genomes increases, new approaches and novel methodology will be needed to develop efficient tools for mining vast amounts of sequence data.

Here, we report a novel yet basic method of defining fast-evolving and slow-evolving genes based on nonsynonymous substitution rates (Ka) in different subgroups or lineages of mammals. We first tested different computational models to see if they provided consistent results when defining the evolution rates of diverse gene classes and families. We then identified percentage shared genes (orthologs) among lineages that were calculated based on different methods, and also looked in more detail at their cellular functions and functional pathways. We also examined the relationship between the evolutionary rates and gene expression levels of these genes, using high-coverage genome sequence and transcriptomic data from thirteen vertebrate species, including human [9], chimpanzee [10], orangutan [11], macaque [12], horse [13], dog [14], cow [15], guinea pig, mouse [16], rat [17], opossum [18], platypus [19], and chicken [20]. Our new method not only confirms the results of many previous studies, but also provides a new and straightforward approach to understanding the evolutionary dynamics of mammalian genes.

## Results and Discussion

### Data and quality control

To examine the divergence between humans and other species, we calculated identities by averaging all orthologs in a species: chimpanzee - 99.23%; orangutan - 98.00%; macaque - 96.09%; horse - 89.44%; dog - 87.93%; cow - 87.36%; guinea pig - 85.91%; mouse - 84.54%; rat - 83.92%; opossum - 77.64%; platypus - 74.37%; and chicken - 72.87%. The data gave rise to a bimodal distribution in overall identities, which distinctly separates highly identical primate sequences from the rest (Additional file 1: Figure 1SA). For quality assessment, we also evaluated the alignment qualities of all orthologs.

First, we found that the number of Ns (uncertain nucleotides) in all coding sequences (CDS) fell within reasonable ranges (mean ± standard deviation): (1) the number of Ns/the number of nucleotides = 0.00002740 ± 0.00059475; (2) the total number of orthologs containing Ns/total number of orthologs × 100% = 1.5084%. Second, we evaluated parameters related to the quality of sequence alignments, such as percentage identity and percentage gap (Additional file 1: Figure S1). All of them provided clues for low mismatching rates and limited number of arbitrarily-aligned positions.

### Indexing evolutionary rates of protein-coding genes

**Figure 1:**
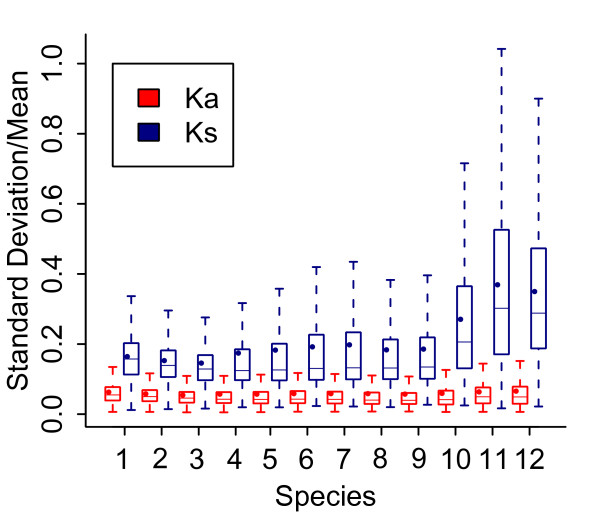
**Divergence index (standard deviation/mean) of Ka and Ks determined based on the eight different methods from the twelve vertebrate species**. In the boxplots, lower quantile, median, and upper quantile were represented in the boxes. Mean values were depicted in dots. Outliers were removed to make the plot straightforward. The number codes for the vertebrate species are: 1, chimp; 2, orangutan; 3, macaque; 4, horse; 5, dog; 6, cow; 7, guinea pig; 8, mouse; 9, rat; 10, opossum; 11, platypus; and 12, chicken.

**Figure 2:**
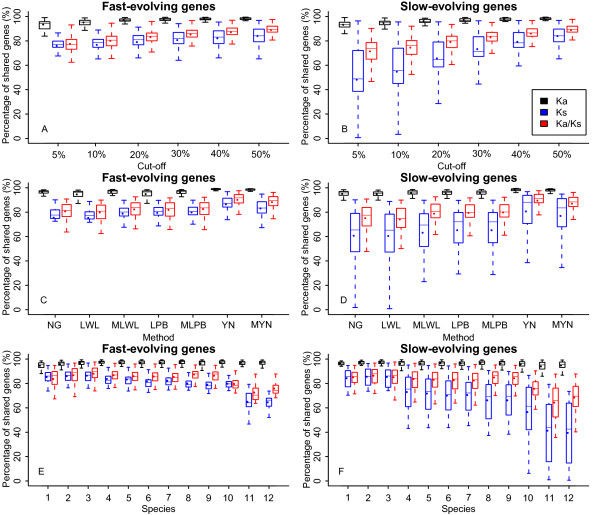
**The percentage of shared genes of Ka, Ks and Ka/Ks based on GY compared with other seven methods in terms of cut-off (A, B), method (C, D), and species (E, F)**. Outliers were removed to make the plots straightforward. The number codes for the species are the same as what in Figure 1.

Ka and Ks are nonsynonymous (amino-acid-changing) and synonymous (silent) substitution rates, respectively, which are governed by sequence contexts that are functionally-relevant, such as coding amino acids and involving in exon splicing [21]. The ratio of the two parameters, Ka/Ks (a measure of selection strength), is defined as the degree of evolutionary change, normalized by random background mutation. We began by scrutinizing the consistency of Ka and Ks estimates using eight commonly-used methods. We defined two divergence indexes: (i) standard deviation normalized by mean, where eight values from all methods are considered to be a group, and (ii) range normalized by mean, where range is the absolute difference between the estimated maximal and minimal values. In order to keep our comparison unbiased, we eliminated gene pairs when any NA (not applicable or infinite) value occurred in Ka or Ks. We observed that the divergence indexes of Ka were significantly smaller than those of Ks in all examined species (P-value < 2.2e-16, Wilcoxon rank sum test) (Figure 1). The result of our second defined index appeared to be very similar to the first (data not shown). We also investigated the performance of these methods in calculating Ka, Ks, and Ka/Ks. First, we considered six cut-off points for grouping and defining fast-evolving and slow-evolving genes: 5%, 10%, 20%, 30%, 40%, and 50% of the total (see Methods). Second, we applied eight commonly-used methods to calculate the parameters for twelve species at each cut-off value. Lastly, we compared the percentage of shared genes (the number of shared genes from different methods, divided by the total number of genes within a chosen cut-off point) calculated by GY and other methods (Figure 2). We observed that Ka had the highest percentage of shared genes, followed by Ka/Ks; Ks always had the lowest. We also made similar observations using our own gamma-series methods [22,23] (data not shown). It was quite clear that Ka calculations had the most consistent results when sorting protein-coding genes based on their evolutionary rates. As the cut-off values increased from 5% to 50%, the percentages of shared genes also increased, reflecting the fact that more shared genes are obtained by setting less stringent cut-offs (Figure 2A and 2B). We also found a rising trend as the model complexity increased in the order of NG, LWL, MLWL, LPB, MLPB, YN, and MYN (Figure 2C and 2D). We examined the impact of divergent distance on gene sorting using the three parameters, and found that the percentage of shared genes referencing to Ka was consistently high across all twelve species, while those referencing to Ka/Ks and Ks decreased with increasing divergence time between human and other studied species (Figure 2E and 2F). In addition, the percentage of shared genes of Ka/Ks remains moderate between those of Ka and Ks. In particular, there should be more variations in the percentages of shared genes determined by Ka/Ks and Ks than by Ka, when we define slow-evolving genes (Figure 2B, D, and 2F). We found consistent results from the various methods when Ka was used as the measure for sorting genes.

The methods used in this study cover a wide range of mutation models with different complexities. NG gives equal weight to every sequence variation path [24] and LWL divides the mutation sites into three categories—non-degenerate, two-fold, and four-fold sites—and assigns fixed weights to synonymous and nonsynonymous sites for the two-fold degenerate sites [25]. LPB adopts a flexible ratio of transitional to transversional substitutions to handle the two-fold sites [26,27]. MLWL or MLPB are improved versions of their parental methods with specific consideration on the arginine codons (an exceptional case from the previous method) [28]. In particular, MLWL also incorporates an independent parameter, the ratio of transitional to transversional substitution rates, into the calculation [28]. Both YN and GY capture the features of codon usage and transition/transversion rates, but they are approximate and maximum likelihood methods, respectively [29,30]. MYN accounts for another important evolutionary characteristic—differences in transitional substitution within purines and pyrimidines [31]. Although these methods model and compute sequence variations in different ways, the Ka values that they calculate appeared to be more consistent than their Ks values or Ka/Ks. We proposed the following reasons (which are not comprehensive): first, real data from large data sets are usually from a broader range of species than computer simulations in the training sets for methodology development, so deviations in Ks values may draw more attentions in discussions. Second, the parameter-rich approaches—such as considering unequal codon usage and unequal transition/transversion rates—may lead to opposite effects on substitution rates when sequence divergence falls out of the "sweet ranges" [25,30,32]. Third, when examining closely related species, such primates, one will find that most Ka/Ks values are smaller than 1 and that Ka values are smaller than Ks values under most conditions. For a very limited number of nonsynonymous substitutions, when evolutionary distance is relatively short between species, models that increase complexity, such as those for correcting multiple hits, may not lead to stable estimations [24,32]. Furthermore, when incorporating the shape parameter of gamma distribution into the commonly approximate Ka/Ks methods, we found previously that Ks is more sensitive to changes in the shape parameter under the condition Ka < Ks [23]. Together, there are stronger influences on Ks than on Ka in two cases: when Ka < Ks and when complexity increases in mutation models. Fourth, it has been suggested that Ks estimation does not work well for comparing extremes, such as closely and distantly related species [33,34]. Occasionally, certain larger Ka/Ks values, greater than 1, are identified, as was done in a comparative study between human and chimpanzee genes, perhaps due to a very small Ks [34].

We also wondered what would happen when Ka becomes saturated as the divergence of the paired sequences increases. Looking at human vs. chicken, we found that the median Ka exceeded 0.2 and that the maximal Ka was as high as 0.6 after the outliers were eliminated (Additional file 1: Figure S2). This result suggested that their Ka values have not approached saturation yet. In addition, we chose the GY method to compute Ka as an estimator of evolutionary rates, since counting methods usually yield more out-of-range values than maximum likelihood methods (data not shown).

### Function characterization of fast-evolving and slow-evolving genes

To learn about the functions of fast-evolving and slow-evolving genes in each species and lineage, we used custom-designed scripts to assess the enrichment of molecular functions (MF), biological processes (BP), and signal/metabolic pathways (Table 1). We noticed that the number of enriched functions related to slow-evolving genes was 2.53 times greater than those related to fast-evolving genes. Fast-evolving genes also had more lineage-specific functions than slow-evolving genes.

**Table 1:** Selected common functional categories of fast-evolving genes and/or slow-evolving genes among mammalian genomes and lineages.

Fast-evolving genes												
Classification	1	2	3	4	5	6	7	8	9	10	11	12
Immunity and defense	*	*	*	*	*	*	*	*	*	*	*	*

Biological process unclassified	*	*	*	*	*	*	*	*	*	*	*	*

Immunoglobulin receptor family member	*	*	*	*	*	*	*	*	*	*	*	*

Defense/immunity protein	*	*	*	*	*	*	*	*	*	*	*	*

Molecular function unclassified	*	*	*	*	*	*	*	*	*	*	*	*

Receptor	*	*		*	*	*	*	*		*		*

Cytokine and chemokine mediated signaling pathway				*	*	*	*	*	*	*	*	*

Cytokine receptor				*	*	*	*	*	*	*	*	*

Ligand-mediated signaling				*	*	*	*	*	*	*	*	

Other defense and immunity protein				*	*	*	*	*	*	*	*	

Natural killer cell mediated immunity	*			*	*	*		*	*	*		

T-cell mediated immunity				*	*	*	*	*	*	*		*

B-cell- and antibody-mediated immunity				*	*	*	*	*	*	*		

Interleukin				*	*	*	*	*	*	*		

Cytokine					*	*	*	*	*	*	*	

**Slow-evolving genes**												

**Classification**	**1**	**2**	**3**	**4**	**5**	**6**	**7**	**8**	**9**	**10**	**11**	**12**

Nucleoside, nucleotide and nucleic acid metabolism	*	*	*	*	*	*	*	*	*	*	*	*

mRNA transcription	*	*	*	*	*	*	*	*	*	*	*	*

Pre-mRNA processing	*	*	*	*	*	*	*	*	*	*	*	*

mRNA splicing	*	*	*	*	*	*	*	*	*	*	*	*

Protein metabolism and modification	*	*	*	*	*	*	*	*	*	*	*	*

Intracellular protein traffic	*	*	*	*	*	*	*	*	*	*	*	*

Nucleic acid binding	*	*	*	*	*	*	*	*	*	*	*	*

mRNA processing factor	*	*	*	*	*	*	*	*	*	*	*	*

mRNA splicing factor	*	*	*	*	*	*	*	*	*	*	*	*

G-protein	*	*	*	*	*	*	*	*	*	*	*	*

Small GTPase	*	*	*	*	*	*	*	*	*	*	*	*

FGF signaling pathway	*	*	*	*	*	*	*	*	*	*	*	*

Ubiquitin proteasome pathway	*	*	*	*	*	*	*	*	*	*	*	*

Protein biosynthesis	*	*	*	*		*	*	*	*	*	*	

Ribosomal protein	*	*	*	*		*	*	*	*	*	*	

General vesicle transport	*	*		*	*	*	*	*	*	*	*	*

mRNA transcription regulation		*	*	*	*	*	*	*	*	*	*	*

Transcription factor		*	*	*	*	*	*	*	*	*	*	*

Huntington disease		*	*	*	*	*	*	*	*	*	*	*

Select regulatory molecule		*		*	*	*	*	*	*	*	*	*

Translation factor		*		*	*	*	*	*	*	*	*	

T cell activation			*	*	*	*	*	*	*	*	*	*

Cell cycle		*		*	*	*	*	*	*	*		*

Mitosis		*		*	*	*	*	*	*	*		

Translation initiation factor		*		*	*	*	*	*	*	*		

Wnt signaling pathway			*	*	*	*	*	*	*	*		*

PDGF signaling pathway				*	*	*	*	*	*	*	*	*

Other transcription factor	*		*	*		*	*	*	*			

Developmental processes				*	*	*	*	*	*	*		

Endocytosis				*	*	*	*	*	*	*		*

Protein phosphorylation				*	*	*	*	*	*		*	*

Hedgehog signaling pathway			*	*	*		*	*	*	*		

Ionotropic glutamate receptor pathway			*			*	*	*	*	*	*	*

Metabotropic glutamate receptor group III pathway			*			*	*	*	*	*	*	*

Parkinson disease			*			*	*	*	*	*	*	*

B cell activation			*			*	*	*	*	*	*	

Angiogenesis				*	*	*	*	*	*	*		*

Ras Pathway				*	*	*	*	*	*	*		*

Note: The asterisks depict function classification of genes in species and lineages, which are significantly enriched based on Fisher's Exact Test after multiple corrections. The species are coded in numbers as what listed in Figure 1.

We found that fast-evolving genes were enriched in immunity-related functions (Table 1), which included genes present in NK, T, and B cells. The genes in NK cells were related to innate immunity (non-specific), and genes in T and B cells were associated with acquired immunity (specific) [35]. Other enriched immunity-related categories of fast-evolving genes included immunoglobulin, cytokine, chemokine, and interleukin. Fast-evolving genes were also enriched in signaling pathways, such as receptors and ligands. Finally, there were a significant number of fast-evolving genes classified as having unknown functions—unclassified biological processes and unclassified molecular functions. It is not surprising that fast-evolving genes may quickly diminish their homology to known proteins and are associated with dietary adaptation, language, appearance, behavior or upright-walking [36]. In the enriched functions of slow-evolving genes, we found a number of important house-keeping functional classes, including transcription, mRNA processing/splicing, translation, protein modification, metabolism, protein traffic, cell cycle, development and endocytosis (Table 1). As a result, fast-evolving and slow-evolving genes have significantly different functions in mammals.

Another point of interest is that we identified two immunity-related function categories, T cell and B cell activation, in the enriched functions of slow-evolving genes (Table 1). We also discovered that immunity-related fast-evolving genes were mostly receptors, ligands, cytokines, and CD (cluster of differentiation) molecules, and that slow-evolving immunity-related genes were usually kinases or adaptor proteins. Taking the human-rat comparison as an example, the receptors included MS4A2, FCER1G, FCGRT, KLRG2, IL1RN, TNFRSF1A, TNFRSF25, IFNGR1, IL2RA, TNFRSF4 and TNFRSF8; the cytokines were IL12A and IL1F9; and the ligands were CCL27 and ICOSLG. All of these are highly conserved, functionally important, and involved in complex immunity-related pathways. Cytokines are also involved in the transfer of information between cells, the regulation of cell physiological processes, and the strengthening of immune-competence [37]. CD proteins, generated during the differentiation of lymphocytes, are a class of cell surface molecules that are recognized by specific antibodies on the surfaces of lymphocytes [38]. Adaptor proteins and kinases play significant roles in signal transduction in cell immune systems, mediate specific interactions between proteins, and activate phosphorylation of the target proteins to functionally modify protein structure and activity [39,40]. In summary, receptors, ligands, cytokines, and CDs are likely to evolve faster than kinases and adaptor proteins, although they all function in the acquired immune system (B cell and T cell immunity). These observations suggest that: (1) Genes in the upstream of the immune-related pathways tend to evolve faster than those in the downstream. (2) Immunity-specific genes are likely to evolve faster than multifunctional house-keeping genes, which also perform fundamental functions in non-immune pathways. (3) Genes encoding for proteins that participate in extracellular communion or the reorganization of external pathogens seem to evolve faster than those which encode proteins that play roles in signal transduction and effector activation within single cells [41]. Similar observations have been reported about the evolution of Drosophila's innate immune system [42].

In addition, we discovered a few enriched functions that were related to neuro-degenerative diseases or nervous system functionality (Table 1). These slow-evolving genes play roles in progressive neuro-degenerative genetic diseases [43], neural-tube defects [44], proliferative disorders in the central nervous system [45], progressions of brain cancers [46,47], and electrical movement within synapses in the brain [48]. These results are consistent with a previous observation that brain-specific genes tend to have relatively low evolutionary rates in mammals [49]. Brain-specific genes may be expressed in multiple distinct neuronal cell types and in a way resemble house-keeping genes in terms of shared cell types.

### Comparisons of fast-evolving and slow-evolving genes and their functions among mammalian lineages

**Figure 3:**
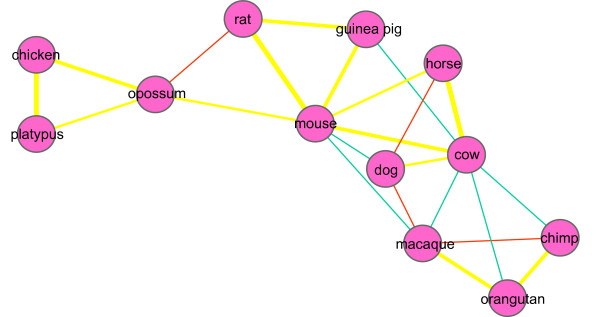
**A network of fast-evolving and slow-evolving genes among twelve mammalian species**. For any two given species, we calculated the shared number of fast-evolving or slow-evolving genes and subsequently divided them based on the total shared number of genes to normalize the correlation coefficients. We connected the species based on the largest two correlation coefficients for each pair. Red and green lines stand for fast-evolving and slow-evolving genes, respectively, and the yellow lines are the sum of both.

We used a network to display the percentages of shared genes among fast-evolving and slow-evolving genes between pairs of mammals (Figure 3). First, two primitive mammals (opossum and platypus) and one bird (chicken) are clearly distinct from other mammals. Second, primates are also closely clustered with one another. Third, mouse serves as an excellent hub that links cow, horse, guinea pig, rat, and opossum. Fourth, large mammals are well connected when all elements are considered. Fifth, some connections may be coincidental, for example, fast-evolving genes shared by dog, horse, and macaque as well as slow-evolving genes shared by cow, macaque, orangutan, and chimp.

We then investigated the exclusive functions of fast-evolving and slow-evolving genes in three mammalian lineages: primates (chimp, orangutan, and macaque), large mammals (horse, dog, and cow), and rodents (guinea pig, mouse, and rat; Table 2, 3, and 4). Although primates are also large mammals, we considered them to be a separate category in order to further stratify our pool. First, we found specific functions that were unique to the three mammalian subgroups in fast-evolving genes: sensory-related (chemosensory perception, olfaction and sensory perception) and cancer related (oncogenesis) in primates (Table 2), immune related (interleukin receptor) in rodents (Table 4), and reproduction related (fertilization) and steroid hormone related (steroid hormone metabolism; Table 3) in large mammals. The first two observations we made are consistent with a previous study [7], and the last one is novel, which may be related to domestication for fast-growth. Second, we also found some lineage-specific functions that involved slow-evolving genes. For instance, we categorized calcium binding proteins, calmodulin related proteins and mitochondrial transport in primates, as well as G protein signalling, enkephalin release, actin binding cytoskeletal proteins, the microtubule family, and exocytosis in rodents. Three critical hormones (alpha adrenergic receptor signalling, oxytocin receptor mediated signalling, and thyrotropin-releasing hormone receptor signalling pathways) are specific to large mammals.

**Table 2:** Functional enrichment of fast-evolving and slow-evolving genes in primates.

Fast-evolving genes				
**ID**	**Category Name**	**chimp**	**orangutan**	**macaque**

BP00183	Chemosensory perception	*	*	
BP00184	Olfaction	*	*	
MF00002	G-protein coupled receptor	*		
BP00182	Sensory perception	*		
BP00281	Oncogenesis	*		
BP00283	Other oncogenesis	*		
MF00175	Major histocompatibility complex antigen	*		
MF00198	Structural protein	*		
MF00256	Intermediate filament	*		
BP00124	Cell adhesion			*

**Slow-evolving genes**				

BP00114	MAPKKK cascade	*		
MF00001	Receptor	*		
MF00113	Phosphatase	*		
BP00029	Lipid and fatty acid binding	*		
BP00134	Mitochondrial transport		*	
MF00188	Select calcium binding protein		*	
MF00218	Calmodulin related protein		*	
MF00079	Other chaperones		*	
MF00098	Large G-protein			*
P00052	TGF-beta signaling pathway			*

Note: The asterisks depict functional classifications of genes that are significantly enriched based on Fisher's Exact Test after multiple corrections among primates.

**Table 3:** Functional enrichment of fast-evolving and slow-evolving genes in large mammals.

Fast-evolving genes				
**ID**	**Category Name**	**horse**	**dog**	**cow**

BP00299	Steroid hormone metabolism	*		
MF00291	Other enzyme activator	*		
MF00247	Membrane-bound signaling molecule		*	*
BP00240	Fertilization			*
BP00274	Cell communication			*

**Slow-evolving genes**				

MF00170	Ligase	*	*	
MF00283	Ubiquitin-protein ligase	*	*	
MF00225	Other zinc finger transcription factor	*		
MF00222	Zinc finger transcription factor		*	
MF00031	Voltage-gated ion channel			*
MF00213	Non-receptor serine/threonine protein kinase			*
P00019	Endothelin signaling pathway			*
P00059	p53 pathway	*		
P00002	Alpha adrenergic receptor signaling pathway			*
P00003	Alzheimer disease-amyloid secretase pathway			*
P04374	5HT2 type receptor mediated signaling pathway			*
P04391	Oxytocin receptor mediated signaling pathway			*
P04394	Thyrotropin-releasing hormone receptor signaling pathway			*

Note: The asterisks depict functional classifications of genes that are significantly enriched based on Fisher's Exact Test after multiple corrections among large mammals.

**Table 4:** Functional enrichment of fast-evolving and slow-evolving genes in rodents.

Fast-evolving genes				
**ID**	**Category Name**	**guiniea pig**	**mouse**	**rat**

MF00250	Serine protease inhibitor	*	*	*
MF00006	Interleukin receptor		*	*
BP00117	JAK-STAT cascade		*	*

**Slow-evolving genes**				

MF00261	Actin binding cytoskeletal protein	*	*	*
MF00264	Microtubule family cytoskeletal protein	*	*	*
MF00009	Glutamate receptor	*	*	
MF00008	Ligand-gated ion channel	*	*	
MF00024	Ion channel	*	*	
MF00069	Ribonucleoprotein	*	*	
BP00051	mRNA end-processing and stability	*		
BP00126	Exocytosis		*	*
BP00246	Ectoderm development		*	*
BP00137	Protein targeting and localization		*	
MF00052	DNA-directed RNA polymerase		*	
MF00156	Other hydrolase		*	
BP00199	Neurogenesis		*	*
BP00133	Nuclear transport			*
BP00066	Protein acetylation			*
P05911	Angiotensin II-stimulated signaling through G proteins and beta-arrestin		*	*
P00007	Axon guidance mediated by semaphorins		*	*
P05731	GABA-B receptor II signaling		*	
P05913	Enkephalin release		*	
P00038	JAK/STAT signaling pathway			*
P05734	Synaptic vesicle trafficking			*

Note: The asterisks depict functional classifications of genes that are significantly enriched based on Fisher's Exact Test after multiple corrections among rodents.

### Comparisons to other studies

There have been three interesting investigations that have used the likelihood ratio test (LRT) to compare two models, and have evaluated the use of Ka/Ks in the identification of positively-selected genes (PSGs) and their enriched functions among six species [6-8]. Our study is unique in that we have analyzed 12 species and considered more than one-thousand fast-evolving genes. The numbers of PSGs in previous studies were at least an order of magnitude less, around tens to hundreds. Although our definition of fast-evolving genes is not fully identical to those of previous studies, our findings on immune-related functions in most species are consistent with previous studies [6,7]. Two other categories that are shared among these studies are chemosensory perception, olfaction, and sensory perception in the human-vs-chimpanzee-specific functions (Table 2) and fertilization in the human-vs-cow-specific functions. This validated the fact that the methods, which were based on simple comparison, yielded conclusions that were similar to those of complicated and over-parametric methods.

Lopez-Bigas et al. conducted a comprehensive study of functional protein sequence divergences between human and other organisms [50]. They focused on variations at the protein level and in a wide range of evolutionary distance, whereas we have focused on variations among mammals at the DNA level [50]. Natural selection acts at three essential levels: domains, catalytic centers, and the DNA and protein level that consists of sequences and protein structures composed of motifs [32]. Since nucleotide sequences are more variable than protein sequences and structures, DNA variations are usually used for the study of short-term evolution, and the latter two are used to study long-term evolution. In our study, we found that the major classified functions were regulatory (e.g. receptor)/response to the surroundings (e.g. immunoglobulin receptor family member) among fast-evolving genes, and metabolism (e.g. protein metabolism and modification), transport (e.g. general vesicle transport) and cell structure (e.g. protein biosynthesis) among slow-evolving genes [50]. We also found developmental processes to be a major functional category in mammals based on the slow-evolving genes when regarding chicken as a reference. This finding agrees with a previous conclusion that development-related genes are highly conserved only among mammals [50]. In addition, at the DNA level, both B-cell-mediated and antibody-mediated immunity and B-cell activation were only identified in mammals but not in chickens. This may reflect differences in B-cell-associated  hormonal responses between the bursa of fabricius unique to birds and the bone  marrow of mammals [51].

### The relationship between evolutionary rate and expression level

**Figure 4:**
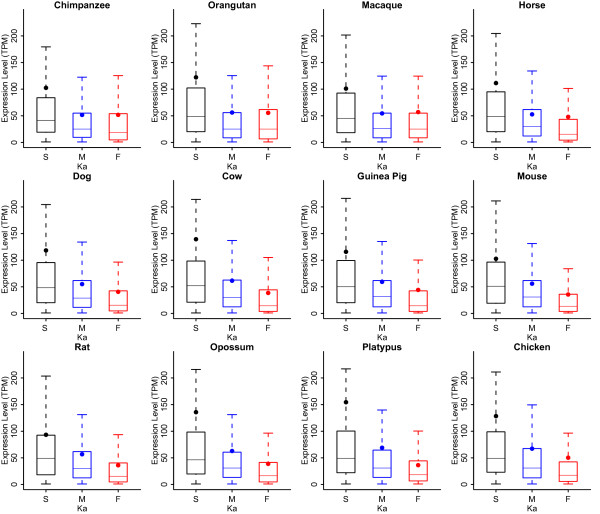
**Expression level correlations and evolvability**. S, M, and F stand for slow-evolving, intermediately-evolving, and fast-evolving genes, respectively. Expression levels were calibrated as the number of transcripts per million (TPM). Outliers were removed to make the plots straightforward.

Our study focused on general expression profiles based on EST data from 18 human tissues (Figure 4). The expression levels of slow-evolving genes appeared to be significantly higher than those of fast-evolving genes (P-value < 2.2e-16, Wilcoxon rank sum test). We also observed that the expression levels of intermediately-evolving genes were significantly higher than those of fast-evolving genes in most species, except for orangutan and macaque. In addition, we found that the mean of gene expressions was always greater than the median, suggesting that most genes are expressed at very low levels and only a small fraction of genes are expressed at high levels [52]. These observations suggest that there is an inverse relationship between gene evolutionary rates and gene expression levels in mammals, which is similar to a previous result reported for the yeast genome [53,54]. House-keeping [55,56], highly-expressed, and old genes [57,58] all tend to evolve slowly [59], and these genes are functionally well-connected and resistant to sequence changes (negative selected). Tissue-specific [55,56], lowly-expressed, and new genes [57,58] tend to evolve quickly; they are often selection-relaxed and evolving toward novel functions. For example, certain immune-related genes always evolve faster to cope with new or multiple pathogen attacks.

### The shared fast-evolving and slow-evolving genes among mammals

To understand the functional relevance common for the fast-evolving or slow-evolving genes among different subgroups of mammals, we categorized the shared genes in the lineages of primates, large mammals, and rodents. There were 185, 609, and 695, fast-evolving genes in primates, large mammals, and rodents, respectively, and 355, 600, and 730 slow-evolving genes. However, we only found 15 fast-evolving and 72 slow-evolving genes that were shared by all nine species. This result suggests that fast-evolving and slow-evolving genes tend to be clade-, lineage- or species-specific. However, a limited numbers of shared genes may still lead to a significant number of shared functions (Table 1).

**Figure 5:**
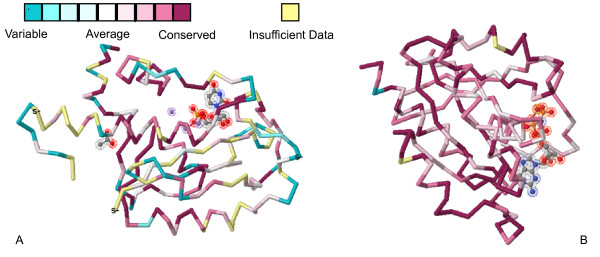
**Three-dimensional conservation grading of ISG20 (A) and RAB30 (B)**. Two 3-D backbone structures of ISG20 and RAB30 were retrieved from PDB code 1WLJ and 2EW1, respectively. (A) The putative conservation grading was based on the alignment of twenty mammalian protein sequences from: Human (*Homo sapiens*), Chimpanzee (*Pan troglodytes*), Orangutan (*Pongo pygmaeus*), Gorilla (*Gorilla gorilla*), Macaque (*Macaca mulatta*), Cow (*Bos taurus*), Dog (*Canis familiaris*), Horse (*Equus caballus*), Cat (*Felis catus*), Guinea Pig (*Cavia porcellus*), Mouse (*Mus musculus*), Rat (*Rattus norvegicus*), Megabat (*Pteropus vampyrus*), Microbat (*Myotis lucifugus*), Pika (*Ochotona princeps*), Hyrax (*Procavia capensis*), Tree Shrew (*Tupaia belangeri*), Dolphin (*Tursiops truncatus*), Opossum (*Monodelphis domestica*), Platypus (*Ornithorhynchus anatinus*). (B) These conservation grades were based on the aligned twenty-two mammalian protein sequences from Human (*Homo sapiens*), Cow (*Bos taurus*), Dog (*Canis familiaris*), Guinea Pig (*Cavia porcellus*), Horse (*Equus caballus*), Cat (*Felis catus*), Elephant (*Loxodonta africana*), Macaque (*Macaca mulatta*), Mouse Lemur (*Microcebus murinus*), Opossum (*Monodelphis domestica*), Mouse (*Mus musculus*), Microbat (*Myotis lucifugus*), Pika (*Ochotona princeps*), Platypus (*Ornithorhynchus anatinus*), Rabbit (*Oryctolagus cuniculus*), Chimpanzee (*Pan troglodytes*), Orangutan (*Pongo pygmaeus*), Hyrax (*Procavia capensis*), Megabat (*Pteropus vampyrus*), Rat (*Rattus norvegicus*), Tree shrew (*Tupaia belangeri*), Dolphin (*Tursiops truncatus*). The color bars from the left to the right measure changes from variable to conserved residues. Conservation grading in yellow indicates the residues whose conservation degrees were not supported with sufficient data.

Although we have only compared human genes (as a reference) with those of other mammals, instead of doing pairwise comparisons, our conclusions can still be easily extended to a broader spectrum of mammals, or even other vertebrates. To validate our analyses, we selected two representative proteins, ISG20 and RAB30 (based on orthologs from 20 and 22 mammals, respectively) from 87 shared fast-/slow-evolving genes in nine species to demonstrate their degrees of variation and conservation (Figure 5). The fast-evolving ISG20 (ranked 25, 71, 94, 69, 95, 128, 321, 58, 82, 280 and 423 in chimpanzee, orangutan, macaque, horse, dog, cow, guinea pig, mouse, rat, opossum and platypus, respectively) and the slow-evolving RAB30 (ranked 1, 418, 334, 117, 105, 127, 48, 49, 33, 132 and 446, respectively in chimpanzee, orangutan, macaque, horse, dog, cow, guinea pig, mouse, rat, opossum and platypus, respectively) can be obviously seen from the degree of variability [60]. These two case studies provide a footnote that supports the reliability of our method.

## Conclusions

In this study, we carried out an evolutionary analysis of human protein-coding genes that are shared among mammals. We not only demonstrated that Ka is a useful and stable indicator for studying mammalian gene evolution, but we also revealed that the rate at which a gene evolves is related to its function. In particular, we found enriched immune-related functions in both fast-evolving and slow-evolving genes, and slow-evolving genes were significantly enriched in functions related to the central nervous system. Furthermore, we observed that slow-evolving genes tended to be expressed at higher levels. Our results provide valuable insights for the functional characterization of genes and gene classes in different mammalian lineages.

## Methods

### Data acquisition and quality assessment

The genome data were collected from Ensembl version 53 [61] (http://www.biomart.org/; http://www.ensembl.org/): human (NCBI36), chimpanzee (CHIMP2.1), orangutan (PPYG2), macaque (MMUL1), horse (EquCab2), dog (BROADD2), cow (Btau4), guinea pig (cavPor3), mouse (NCBIM37), rat (RGSC3.4), opossum (BROADO3), platypus (OANA5), and chicken (WASHUC2). We also collected ortholog sequences of humans and other species, saving only the gene pairs marked as one-to-one match to avoid ambiguous definition of orthologs. We used ClustalW [62] to align human amino acid sequences with those of other species, and then translated them back to their corresponding nucleotide sequences.

### Defining fast-evolving, intermediately-evolving, and slow-evolving genes

We estimated the non-synonymous substitution rates and synonymous substitution rates of orthologs based on a number of algorithms, including NG (the different methods are abbreviated as their authors' last name initials; M stands for a modified version of the original methods) [24], LWL [25], MLWL [28], LPB [26,27], MLPB [28], YN [30], MYN [31], GY [29], and the gamma-series methods [22,23] used in KaKs Calculator 2.0 tool [63]. We adopted 10% as the cut-off value to define fast-, intermediately- or slow-evolving genes in each lineage. We sorted genes by their Ka values from smallest to largest in each lineage, and defined genes corresponding to the lowest, middle, and highest 10% of Ka values to be slow-evolving, intermediately-evolving, and fast-evolving genes, respectively. In this procedure, we considered NA (not applicable) values to be 0, because we observed that NA values are usually associated with 100% identical gene pairs, except in the cases of a few indels (inserts or deletions).

### Functional classification and other analyses

We used IDConvertor [64] to convert the different ID between different gene accessions and utilized the Protein Analysis through Evolutionary Relationships (PANTHER) online system to annotate genes at three levels: biological processes, molecular functions, and pathways [65]. Enrichment analysis was performed based on a combination of Fisher's exact test and multiple testing Bonferroni Step-down (Holm) correction [66]. The cut-off in functional enrichment test is 0.1. The network created based on fast- and slow-evolving genes was drawn with the software Cytoscape [67]. Conserved grade illustrations were created using the Consurf server [68] after submitting protein alignments built with ClustalX [62]. The three-dimensional structures of the corresponding proteins were retrieved from the Protein Data Bank (PDB) [69]. For gene expression analysis, we used the expression profiling of Expressed Sequence Tags (EST) data pooled from 18 tissues as described previously in our published work [70].

## Competing interests

The authors declare that they have no competing interests.

## Authors' contributions

DW and JY conceived and designed this study; DW drafted this paper; DW, FL, LW collected the data; DW and FL analyzed the data; SH participated in revising the paper; JY supervised the project and revised this manuscript. All authors read and approved the final version of the manuscript.

## Reviewers' comments

### Reviewer's report 1

Anamaria Necsulea, Université de Lyon, F-69000, Lyon; Université Lyon 1; CNRS, UMR 5558, Laboratoire de Biométrie et Biologie Evolutive, F-69622, Villeurbanne, France; HELIX, Unité de recherche INRIA (nominated by Nicolas Galtier, CNRS-Université Montpellier II, Laboratoire "Genome, Populations, Interactions, Adaptation", Montpellier, France)

This manuscript attempts to assess the rate of evolution of mammalian protein-coding genes, and to extract the defining characteristics of fast and slow evolving genes. This subject has been addressed extensively in the literature, and the findings of the present manuscript are not novel. Unfortunately, the lack of novelty is not the biggest fault of this article: the methodology employed is often flawed and the text is very badly written.

In order to estimate the rates of evolution of mammalian protein-coding genes, the authors compute the Ka, Ks and Ka/Ks values for pairwise 1-1 orthologues between human and the other species in their dataset. The Ka and Ks computations are performed with several methods available in the literature, and they observe that the Ks measurement does not yield consistent results between the different methods employed. Rather than investigating in detail why this happens (the saturation problem is only briefly mentioned), the authors decide to use Ka as an estimate of the rate of evolution. This is of course correct if the rate of protein sequence evolution is of interest, but without any correction for the mutation rate, one cannot make inferences about the strength of natural selection on protein-coding genes based on Ka alone. Yet the authors use Ka as "an estimator of selection" (page 9).

#### Authors' response

*We added a few discussion points about the reason why Ka values from multiple methods yield more consistent results than Ks values. We also changed the description "an estimator of selection" into "Ka as an estimator of evolutionary rate"*.

The authors then go on to compare the results obtained for the different mammals, and they infer lineage-specific accelerations based solely on the pairwise "human-other species" comparisons. This does not make sense. The authors should be aware that there are methods for the estimation of branch-specific Ka, Ks and Ka/Ks ratios that use a multiple-species sequence alignment and that take into account the underlying phylogeny (see for example PAML — perhaps the most commonly used — Z. Yang, Mol. Biol. Evol., 2007).

#### Authors' response

*We are fully aware that the Likelihood Ratio Test (LRT) methods *[71,72]*are applicable in inferring positive selections on genes in specific braches (or clades) and researchers use these methods to different species including mammals and others *[6-8,73]. *One of the objectives of our study is to compare our method based on simple pairwise comparison between human and other mammals with the LRT methods. We found that our method is simply capable of capturing the key conclusions from other methods and can be used to discover evolutionary features of lineage-specific genes (such as lineage-specific functions of large mammals). Furthermore, pairwise alignments utilize more sequence information than multiple sequence alignments do, especially when closely related (for instance, a few percent differences) and less-than-perfect sequences are aligned. The LRT methods usually require the construction of phylogenies and compare two models, and they are usually parameter-rich, especially when a large number of sequences from multiple species are examined. After all, we are not here to challenge the power of the LRT methods, but to suggest a simple and efficient method as an alternative*.

Finally, the manuscript is very poorly written, to the point that the meaning of the phrases is often incomprehensible. This is evident even for the title: "A method for defining evolving protein-coding genes" — evolving as opposed to what?

#### Authors' response

*We revised the manuscript again for clarity and accuracy. We also changed the title into "A method for defining fast-evolving and slow-evolving protein-coding genes"*.

#### Comments from the second round of reviewing

I am not in the least convinced by the revision of the manuscript. The modifications to the original manuscript are only superficial, and the content remains unworthy of publication. None of the results are new. The analysis of Ka rates is now so well established, that it is generally done in practical courses, for a bachelor's degree, and cannot by itself constitute the subject of a publication. Moreover, the methodology and the interpretation of the results are flawed. The authors continue to perform pairwise comparisons between human and each of the other species, and yet they discuss lineage-specific accelerations. This does not make sense. To give just one example, the authors discuss the proportion of fast-evolving genes that are 'shared among mammals'. Could it be that these genes are in fact accelerated only in the human lineage? When performing pairwise comparisons, with human as a reference, the genes that are specific to human would appear as fast-evolving in all comparisons.

#### Authors' response

*First, what we are emphasizing here is not the ways to calculate Ka and Ks but their overall effects on data analyses, which are useful for the end users, especially biologists who are eager to understand the essence of the methodology and their applications. Second, the calculations for Ka and Ks values are all relative. We have several reasons for choosing just human-to-other-mammal comparisons. The most important reason is the fact that human data are the best among all mammalian genomes sequenced so far. Other mammalian genomes are not sequenced, assembled, and annotated to the standard of human data yet. The net result for choosing a shared ortholog set for all mammals, due to the variable data quality, is that we will not be able to find good representatives for fast-evolving genes that share similar functional categories since most of the gene annotations rely heavily on those of the human data. Especially for extreme cases, such as fast-evolving genes, we do not anticipate that these genes themselves are shared by all or even most of the mammals but do share the specific functional categories. The second reason why we only use human-to-other-mammal comparison is data size. If we did an all-against-all analysis, we would have to write several other manuscripts to describe our results and that would not be desirable either at this point in time: we would have to improve the data quality for all other sequenced mammals, except for human and mouse perhaps, which are better assembled and annotated. The last, but not the least important, reason we have chosen to compare human genes to their orthologs in other mammalian species is so that we can understand the evolution rates of human genes first. In other words, we want to first investigate how human protein-coding genes have evolved from their ancestors in other presumably distinct mammalian lineages. In addition, we carried out a mouse-centric analysis and validated most of the human-centric results in the function categories of fast- or slow-evolving genes (Additional file *1*: Table S1)*.

### Reviewer's report 2

Subhajyoti De, Dana-Farber Cancer Institute and Harvard School of Public Health, Harvard University, Boston, USA (nominated by Sarah Teichmann, MRC Laboratory of Molecular Biology, Cambridge, United Kingdom).

The paper 'A method for defining evolving protein coding genes' by Wang et al. presents an evolutionary analysis of orthologus protein-coding genes across different species. My main concern with this paper is the lack of novelty. The main conclusions of this paper — (i) different functional classes of genes evolve differently, (ii) highly expressed genes evolve slowly and (iii) fast evolving genes often evolve in a lineage-specific manner—have already been reported comprehensively by several groups (Gerstein, Siepel, Hurst, Koonin, Drummond, Nielsen, Bustamante and many other labs). The authors merely reconfirm their findings. Many of those previous papers are not cited either.

#### Authors' response

*As pointed out by Dr. Claus O. Wilke, we do have a "central hypothesis" here, which is novel and valid. We are not contradicting any of the conclusions made by many others who have applied the methods we used also to analyze mammalian genomes or any other multiple sequences, but merely share our surprise that Ka calculation is unusually robust among all these methods. Nevertheless, we added more citations in the revised version as we made further comparisons with several representative publications*.

I am also confused with the other conclusion of this paper — 'Ka is better than Ka/Ks and Ks for evolutionary estimation'. Ka, Ks and Ka/Ks quantify different evolutionary features, and it would be unfair to compare them directly.

#### Authors' response

*We revised the sentence and it is now reads: "Ka estimated from a diverse selection of methods has more consistent results than Ka/Ks and Ks*.

In addition, many statements in that section are incorrect. For instance,

(i) "Ka/Ks and Ka are usually used to weigh the evolutionary rate for large number of genes, where the former has been used more frequently." — Ka/Ks is a measure of selection, and not used to calculate evolutionary divergence per se.

#### Authors' response

*We have revised this sentence accordingly*.

(ii) "We decided to choose Ka, an estimator of selection, rather than Ks, an indicator of random mutations for our studies" — Ka is a measure of nonsynonymous divergence and not a measure of selection. Moreover, Ks is often influenced by sequence context (see papers by Laurence Hurst in 2007).

#### Authors' response

*We have revised the sentences and added the citation accordingly*.

(iii) "Occasionally, larger Ka/Ks values, greater than 1, have been identified, such as those in a comparative study between human and chimpanzee, perhaps due to smaller Ks (Koonin and Rogozin, 2003)" — the statement, and the paragraph, lead to an incomplete impression that all Ka/Ks > 1 in human-chimpanzee are due to small Ks and therefore not indicative of selection. Yes, it is possible that for some genes high Ka/Ks can arise by chance, but that's not the complete picture. Many genes with high Ka/Ks ratio are classic examples of positive selection (e.g. FOXP2, and also see Clark et al. Science, 2003 [8], Nielsen et al. in PLoS Biol. 2005 [6]).

#### Authors' response

*We have revised the sentences accordingly*.

Lopez-Bigas et al. studied evolution of human protein coding genes in different eukaryotes ranging from primates and other mammals to yeast at the protein sequence level. They also showed that sequence similarity and Ka (or dN) are highly correlated (see supplementary information of Lopez Bigas et al.[50]). Therefore it is not surprising that using Ka, the authors find similar results.

#### Authors' response

*Lopez-Bigas et al found a negative correlation (nearly -0.7) between Conservation Score (CS) and Ka *[50]. *This linearly correlated relationship does not mean that the two indexes are exactly the same. As matter of fact, the same protein may be encoded by different codons at the nucleotide level. Therefore, the calculations of protein similarity and nonsynonymous substitution rates (nonsynonymous substitutions/nonsynonymous sites) on the basis of nucleotide substitution models may lead to different results. In addition, we did find some new functions at the DNA level (e.g. B cell- and antibody-mediated immunity as well as B-cell activation)*.

Please note that Gene and ortholog annotation have improved since Ensembl v53 (especially for chimpanzee, orang etc). Moreover, gene expression data for over 70 tissue types in both human and mouse are available from GNF-Symatlas, and it is pretty comprehensive.

#### Authors' response

*We are grateful to the reviewer for the note. Actually, at the time we began this project, Ensembl version 53 (released in 2009) was the most up-to-date. We did check the newer versions and the methodology used for database construction has not been changed. The only things that have changed are a few up-to-date genome assemblies which will only result in incremental improvements on a negligible fraction of the genes that we analyzed here. We used previously published procedures to select Expressed Sequence Tag (EST) data from 18 representative tissues (referring to major anatomic systems and succeeded in applying the data to define housekeeping genes *[56,70]*and minimal introns related studies *[74]. *It is rather unfortunate that the current RNA-seq data have not covered enough tissue samples yet. In addition, the house-keeping genes we defined seem holding very well in our recent analysis with limited number of tissue samples (around 10; data not shown)*.

The authors calculated Ka, Ks, Ka/Ks using several different algorithms and found that results do not exactly overlap i.e. shared gene ratio is not 100%. Perhaps it would be interesting to evaluate the performance of those algorithms, check which ones provide more consistent results and why.

#### Authors' response

*In the computer simulations of our previous studies, we have found that the Ka/Ks-calculating methods based on similar substitution models (capturing similar evolutionary features) often yielded similar results *[23,75]. *In this study, however, we were surprised to find consistent Ka values from this diverse group of methods. We added new analyses and discussions in the revised manuscript concerning the causative factors of inconsistency between different methods' estimates of Ka and Ks*.

### Reviewer's report 3

Claus O. Wilke, Center for Computational Biology and Bioinformatics and Institute for Cell and Molecular Biology, University of Texas, Austin, Texas, United States

The authors study the evolutionary rates of mammalian genes using eight different methods of evolutionary-rate calculation. They conclude that Ka is more consistently estimated by these different methods than Ks and that therefore Ka will be more informative in many contexts than Ks or Ka/Ks.

While I think that the paper makes a valuable contribution, I feel that the impact of the paper has been diluted by the authors' choice to actually combine two separate parts (with separate messages) into one paper. The first part (which I find valuable) is the analysis of the consistency of rate estimations by different methods. The second part (of whose value I'm less convinced) looks at the functional classification of genes evolving at different rates.

#### Authors' Response

*The point is well-taken. In the second part, we just showed selective examples (maybe just the tip of the iceberg) for possible applications of the method. We have weakened some of our conclusions in the second part and explained the weakness of the data set itself (see response to the Reviewer 1). We are in the process of doing thorough analysis on genes that are classified based on Ka values among mammalian genomes, and pinpointing their functional roles in gene interaction networks*.

Specific comments:

1. The first part is improved in the revision, but still not entirely satisfying. I don't really get a good take-home message from this part. Which method should I use to estimate evolutionary rates? Are there specific reasons why some methods give different results than others? Maybe the differences in Ks results simply reflect improvements in estimation methods over time? Note that the model abbreviations (NG, LWL, MLWL, etc) are never defined.

#### Authors' response

We continue to improve our writing in the current revision. The take-home messages for the first part are two-fold. First, Ka calculation is more consistent than Ks calculation regardless of what methods are used. Second, depending on the evolutionary distance between the sequences of the two species evaluated, one can choose more or less complex models for Ka and Ks calculation but they result in more or less similar results for Ka but not for Ks. The reasons why Ks values vary when using different methods are complicated, as we have discussed in the manuscript. We added a note to explain the naming conventions for the different methods.

2. I remain unconvinced by the second part. My most important criticism, that the functional characterization is confounded by expression level, has not been substantially addressed.

#### Authors' response

*We cited 8 consecutive references (from 52 to 59) where this issue has been intensively discussed*.

3. I'm not convinced that the title faithfully reflects the contents of the paper. What is the method for defining fast-evolving and slow-evolving protein-coding genes? If the method is simply "Use Ka", I'd argue that people have done that before.

#### Authors' response

*We have changed the title to "Nonsynonymous substitution rate (Ka) is a relatively consistent parameter for defining fast-evolving and slow-evolving protein-coding genes". We have searched the related literature carefully and have not found publications that have done such thorough evaluations on the methods*.

## Supplementary Material

Additional file 1 **Estimation of the sequence alignment quality (Figure S1), boxplots of Ka distributions in twelve species (Figure S2) and selected common functional categories of fast-evolving and slow-evolving genes based on mouse-centric analyses (Table S1)**. Click here for file
