# Supplementary material for: Nonsynonymous substitution rate (Ka) is a relatively consistent parameter for defining fast-evolving and slow-evolving protein-coding genes
Source: Biol Direct. 2011 Feb 22;6:13. doi: 10.1186/1745-6150-6-13 (PMC3055854; doi:10.1186/1745-6150-6-13)
Supplement: Additional file 1 — Estimation of the sequence alignment quality (Figure S1), boxplots of Ka distributions in twelve species (Figure S2) and selected common functional categories of fast-evolving and slow-evolving genes based on mouse-centric analyses (Table S1). [file 1745-6150-6-13-S1.PDF]

Additional file 1

Figure S1. Quality evaluation of pooled aligned data from twelve species: (A) Distribution of identity% (=identical nucleotide pair number/alignment length without consideration of gap length*100%); (B) Distribution of gap% (=gap number/alignment length*100%).

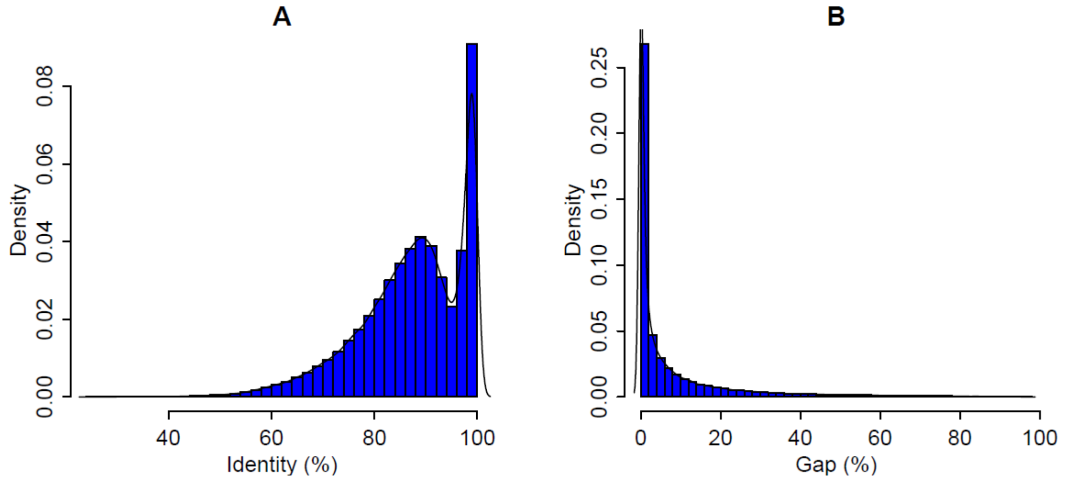

Figure S2. Boxplots of Ka distributions in twelve species.

The number codes for the species and detailed meanings of lines in the boxplots are the same as what in Figure 1. Outliers were removed to make the figure clear.

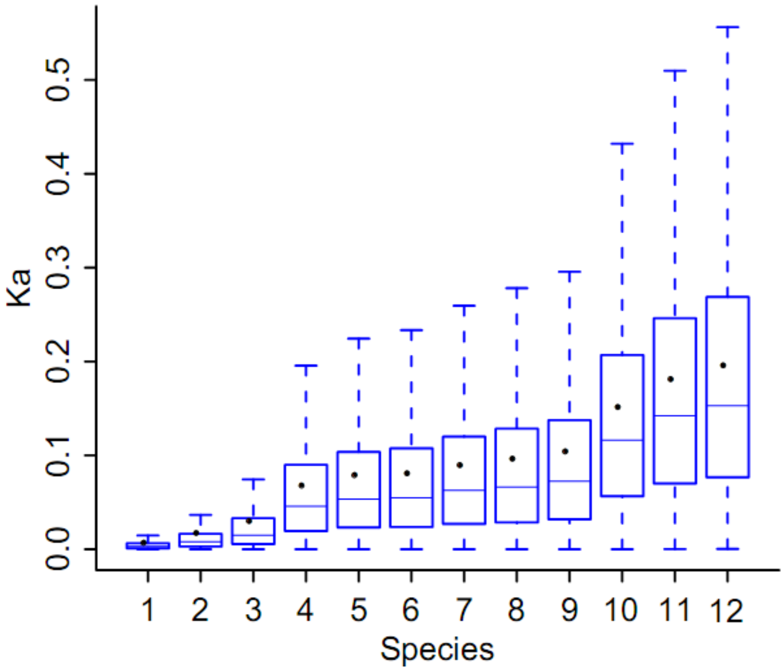

Table S1. Selected common functional categories of fast-evolving genes (A) and slow-evolving genes (B) based on mouse-centric analyses.

A.

Classification	1	2	3	4	5	6	7	8	9	10
B cell mediated immunity	*	*	*		*	*	*	*	*	*
cellular defense response	*	*	*		*	*	*	*	*	*
chemokine	*	*	*		*	*	*	*	*	
cytokine	*	*	*		*	*	*	*	*	*
cytokine receptor	*	*	*		*	*	*	*	*	*
cytokine receptor activity	*	*	*		*	*	*	*	*	*
defense/immunity protein	*	*	*		*	*	*	*	*	*
immune response	*	*	*		*	*	*	*	*	*
immune system process	*	*	*			*	*		*	*
immunoglobulin receptor superfamily	*	*	*		*	*	*	*	*	*
interferon receptor activity	*	*	*			*	*		*	*
macrophage activation	*	*	*			*	*		*	*
natural killer cell activation	*	*	*		*	*	*	*	*	*
response to interferon-gamma	*	*	*		*	*	*	*	*	
response to stimulus	*	*	*		*	*	*	*	*	*
tumor necrosis factor receptor	*	*	*			*	*		*	*
tumor necrosis factor receptor activity	*	*	*			*	*		*	*
type I cytokine receptor	*	*	*		*	*	*	*	*	*
type II cytokine receptor	*	*	*			*	*		*	*

B.

Classification	1	2	3	4	5	6	7	8	9	10
5HT2 type receptor mediated signaling pathway	*	*	*	*	*	*	*	*	*	*
actin cytoskeleton	*	*	*	*	*	*	*	*	*	
actin family cytoskeletal protein	*	*	*	*	*	*	*	*	*	
Alzheimer disease-presenilin pathway	*	*	*	*	*	*	*	*	*	
Angiogenesis	*	*	*	*	*	*	*	*	*	
B cell activation	*	*	*	*	*	*	*		*	*
binding	*	*	*	*	*	*	*	*	*	*
Cadherin signaling pathway	*	*	*	*	*	*	*	*	*	
catalytic activity	*	*	*	*	*	*	*	*	*	*
cell cycle	*	*	*	*	*	*	*	*	*	*
cellular component organization	*	*	*	*	*	*	*	*	*	*
cellular process	*	*	*	*	*	*	*	*	*	*

cytoskeletal protein	*	*	*	*	*	*	*	*	*	
Cytoskeletal regulation by Rho GTPase	*	*	*	*	*	*	*	*	*	*
cytoskeleton	*	*	*	*	*	*	*	*	*	
developmental process	*	*	*	*	*	*	*	*	*	
DNA binding	*	*	*	*	*	*	*	*	*	*
DNA binding protein	*	*	*	*	*	*	*	*	*	*
DNA-directed RNA polymerase	*	*	*	*	*	*	*	*	*	*
DNA-directed RNA polymerase activity	*	*	*	*	*	*	*	*	*	*
EGF receptor signaling pathway	*	*	*	*	*	*	*	*	*	*
endocytosis	*	*	*	*	*	*	*	*	*	
enzyme modulator	*	*	*	*	*	*	*	*	*	
exocytosis	*	*	*	*	*	*	*	*	*	*
FGF signaling pathway	*	*	*	*	*	*	*	*	*	*
G-protein	*	*	*	*	*	*	*	*	*	*
GTPase activity	*	*	*	*	*	*	*	*	*	*
helix-turn-helix transcription factor	*	*	*	*	*	*	*	*	*	*
heterotrimeric G-protein	*	*	*	*	*	*	*	*	*	*
heterotrimeric G-protein complex	*	*	*	*	*	*	*	*	*	*
histone	*	*	*	*	*	*	*	*	*	*
homeobox transcription factor	*	*	*	*	*	*	*	*	*	*
Huntington disease	*	*	*	*	*	*	*	*	*	*
Inflammation mediated by chemokine and cytokine signaling pathway	*	*	*	*	*	*	*	*	*	*
Integrin signalling pathway	*	*	*	*	*	*	*	*	*	*
intracellular	*	*	*	*	*	*	*	*	*	*
intracellular protein transport	*	*	*	*	*	*	*	*	*	*
intracellular signaling cascade	*	*	*	*	*		*	*	*	*
ion channel	*	*	*	*	*	*	*	*	*	*
ion channel activity	*	*	*	*	*	*	*	*	*	*
Ionotropic glutamate receptor pathway	*	*	*	*	*	*	*	*	*	*
ligand-gated ion channel	*	*	*	*	*	*	*	*	*	*
metabolic process	*	*	*	*	*	*	*	*	*	*
Metabotropic glutamate receptor group II pathway	*	*	*	*	*	*	*	*	*	*
Metabotropic glutamate receptor group III pathway	*	*	*	*	*	*	*	*	*	*
mitosis	*	*	*	*	*	*	*	*	*	*
mRNA processing factor	*	*	*	*	*	*	*	*	*	*
Muscarinic acetylcholine receptor 1 and 3 signaling pathway	*	*	*	*	*	*	*	*	*	*
nervous system development	*	*	*	*	*	*	*	*	*	
nuclear transport	*	*	*	*	*	*	*	*	*	*
nucleic acid binding	*	*	*	*	*	*	*	*	*	*
nucleobase, nucleoside, nucleotide and nucleic acid metabolic process	*	*	*	*	*	*	*	*	*	*
Oxytocin receptor mediated signaling pathway	*	*	*	*	*	*	*	*	*	*
Parkinson disease	*	*	*	*	*	*	*	*	*	*
PDGF signaling pathway	*	*		*	*	*	*	*	*	*
PI3 kinase pathway	*	*	*	*	*	*	*	*	*	*

primary metabolic process	*	*	*	*	*	*	*	*	*	*
protein binding	*	*	*	*	*	*	*	*	*	
protein metabolic process	*	*	*	*	*	*	*	*	*	*
protein transport	*	*	*	*	*	*	*	*	*	*
Ras Pathway	*	*	*	*	*	*	*	*	*	*
ribonucleoprotein	*	*	*	*	*	*	*	*	*	
ribonucleoprotein complex	*	*	*	*	*	*	*	*	*	
RNA binding	*	*	*	*	*	*	*	*	*	*
RNA binding protein	*	*	*	*	*	*	*	*	*	*
RNA splicing factor activity, transesterification mechanism	*	*	*	*	*	*	*	*	*	*
small GTPase	*	*	*	*	*	*	*	*	*	*
structural constituent of cytoskeleton	*	*	*	*	*	*	*	*	*	
T cell activation	*	*	*	*	*	*	*	*	*	*
Thyrotropin-releasing hormone receptor signaling pathway	*	*	*	*	*	*	*	*	*	*
transcription factor	*	*	*	*	*	*	*	*	*	*
transcription factor activity	*	*	*	*	*	*	*	*	*	*
transcription regulator activity	*	*	*	*	*	*	*	*	*	*
translation factor	*	*	*	*	*	*	*	*	*	*
translation factor activity, nucleic acid binding	*	*	*	*	*	*	*	*	*	*
translation initiation factor activity	*	*	*	*	*	*	*	*	*	*
translation regulator activity	*	*	*	*	*	*	*	*	*	*
transport	*	*	*	*	*	*	*	*	*	*
Ubiquitin proteasome pathway	*	*	*	*	*	*	*	*	*	*
vesicle-mediated transport	*	*	*	*	*	*	*	*	*	*
Wnt signaling pathway	*	*	*	*	*	*	*	*	*	*

Note: We performed the analyses between mouse genes and their orthologs in other mammals and defined the numbers in the tables as follows: 1, chimp; 2, orangutan; 3, macaque; 4, horse; 5, dog; 6, cow; 7, guinea pig; 8, rat; 9, opossum; 10, platypus. The asterisks depict function classification of genes in species and lineages, which are significantly enriched based on Fisher’s Exact Test after multiple corrections. Due to the usage of an updated version of PANTHER database in this analysis, there may be some category name differences between different analyses.
